# *Yarrowia lipolytica* produces lipid-rich biomass in medium mimicking lignocellulosic biomass hydrolysate

**DOI:** 10.1007/s00253-023-12565-6

**Published:** 2023-05-16

**Authors:** Bruna Dias, Helena Fernandes, Marlene Lopes, Isabel Belo

**Affiliations:** 1 CEB–Centre of Biological Engineering, University of Minho, Campus de Gualtar, 4710-057 Braga, Portugal; 2 LABBELS–Associate Laboratory, Guimarães, Braga Portugal

**Keywords:** *Yarrowia lipolytica*, Lignocellulosic biomass hydrolysates, Microbial lipids, Batch cultures, Bioreactor

## Abstract

**Abstract:**

In recent years, lignocellulosic biomass has become an attractive low-cost raw material for microbial bioprocesses aiming the production of biofuels and other valuable chemicals. However, these feedstocks require preliminary pretreatments to increase their utilization by microorganisms, which may lead to the formation of various compounds (acetic acid, formic acid, furfural, 5-hydroxymethylfurfural, *p*-coumaric acid, vanillin, or benzoic acid) with antimicrobial activity. Batch cultures in microplate wells demonstrated the ability of *Yarrowia* strains (three of *Y. lipolytica* and one of *Y. divulgata*) to grow in media containing each one of these compounds. Cellular growth of *Yarrowia lipolytica* W29 and NCYC 2904 (chosen strains) was proven in Erlenmeyer flasks and bioreactor experiments where an accumulation of intracellular lipids was also observed in culture medium mimicking lignocellulosic biomass hydrolysate containing glucose, xylose, acetic acid, formic acid, furfural, and 5-HMF. Lipid contents of 35% (w/w) and 42% (w/w) were obtained in bioreactor batch cultures with *Y. lipolytica* W29 and NCYC 2904, respectively, showing the potential of this oleaginous yeast to use lignocellulosic biomass hydrolysates as feedstock for obtaining valuable compounds, such as microbial lipids that have many industrial applications.

**Key Points:**

• *Yarrowia strains tolerate compounds found in lignocellulosic biomass hydrolysate*

• *Y. lipolytica consumed compounds found in lignocellulosic biomass hydrolysate*

• *42% (w/w) of microbial lipids was attained in bioreactor batch cultures*

**Graphical Abstract:**

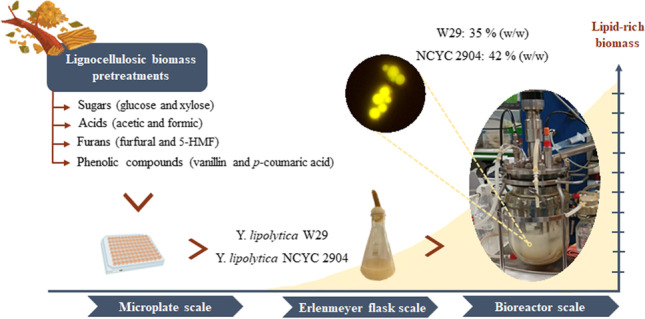

## Introduction

Lignocellulosic biomass (LB) is a low-cost, renewable, and abundant resource (200 billion tons generated per year) that is derived from plants, food, and agro-based industries (Mankar et al. 2021; Prasad et al. 2022). Generally, LB is mainly constituted of cellulose (35–60%), hemicellulose (20–35%), lignin (5–40%), and low quantities of ash and volatile compounds (Zhang et al. 2021).

The rigid and recalcitrance structure of LB is related to the highly polymerized and crystalline cellulose structure, hindering LB’s direct utilization of microbial species (Mankar et al. 2021). Therefore, some preliminary steps are needed to disrupt the cell wall structure, increasing the surface accessibility of cellulose and hemicellulose fractions for enzymatic hydrolysis, resulting in fermentable sugars release (Prasad et al. 2022; Rajesh Banu et al. 2021; Yang et al. 2018). Different pretreatments, such as physical (e.g. milling/grinding, ultrasound, microwave radiation), chemical (e.g. acids, alkalis, ozone, and organic solvents), physicochemical (e.g. ammonia fiber explosion, steam explosion, liquid hot water treatment), and biological methods (e.g. bioabatement and enzymatic treatments) are used to reduce the particle size of LB and/or to cleave lignin, cellulose, and hemicellulose chemical bonds (Caporusso et al. 2021; Jönsson and Martín 2016; Prasad et al. 2022).

Depending on the type of LB, as well as the operational conditions of the pretreatments, some undesirable compounds with antimicrobial activity can be found in LB hydrolysates (LBH) (Jönsson and Martín 2016; Konzock et al. 2021). These compounds are divided into three groups: phenolic compounds, originated from lignin degradation (e.g. syringaldehyde, p-hydroxybenzaldehyde, vanillin, among others); furan aldehydes, produced from sugar dehydration (e.g. furfural and 5-hydroxymethylfurfural (5-HMF)); and weak organic acids, derived from the hydrolysis of acetyl groups and the degradation of furan (e.g. acetic acid, formic acid, levulinic acid, among others) (Caporusso et al. 2021; Wang et al. 2018). To avoid the harmful effects of these LBH-derived compounds (LBH-C), they can be removed and/or chemically modified using physical (e.g. evaporation), chemical (e.g. solvent extraction, use of ion exchange resins), or biological (e.g. specific enzymes targeting toxic compounds) methodologies. Although these procedures may effectively remove LBH-C, they can also result in the substantial loss of fermentable sugars and are not economically advantageous due to the high costs associated with additional processing steps (Parawira and Tekere 2011; Yang et al. 2018). In this sense, an interesting alternative is emerging, focusing on the screening of highly resistant microorganisms capable of growing in LBH-C (Yang et al. 2018). This is a promising, eco-friendly, and cost-effective approach in comparison with other methods since it does not require large equipment, hazardous solvents, and high energy expenditures, resulting in the production of value-added compounds (Prasad et al. 2022).

Currently, the majority of LB is burned or remains unused, while only circa 3% of total LB is efficiently utilized in processes within a circular bioeconomy concept (Rajesh Banu et al. 2021). Hydrolysates of sugarcane bagasse, rice husk, wheat straw, corn cob, oil palm fruit, gardening residues, and rapeseed straw have been studied as valuable feedstocks to produce biofuels, bioenergy (e.g. bioethanol, biogas, biomethane, and biohydrogen), and other value-added products (e.g. lactate, lactic acid, xylitol, succinic acid, phenolic compounds, and pectin) (Haldar and Purkait 2020; Rajesh Banu et al. 2021; Rosales-Calderon and Arantes 2019) in microbial-based bioprocesses. Although these raw materials are low-cost, renewable, and abundant, microbial lipids production by oleaginous yeasts using LB as substrate is still a recent research topic (Rosales-Calderon and Arantes 2019; Valdés et al. 2020).

Since studies focusing on the performance of *Yarrowia* species in the presence of LBH-C are still underexplored, the first objective of this work was to evaluate the effect of acetic acid, formic acid, furfural, 5-HMF, phenol, *p*-coumaric acid, vanillin, and benzoic acid on four *Yarrowia* strains (three of *Y. lipolytica* and one of *Y. divulgata*) grown in 96-well microplate batch cultures. Strains of *Y. lipolytica* selected (W29 and NCYC 2904) were used in Erlenmeyer flasks and bioreactor batch cultures to assess their ability to grow and accumulate intracellular lipids in media containing LBH-C.

## sMaterials and methods

### Yeast strains and maintenance

*Yarrowia lipolytica* W29 (ATCC 20460, isolated from soil), *Y. lipolytica* NCYC 2904 (isolated from a maize-processing), *Y. lipolytica* Ch 1/5 (isolated from cheese) (Nagy 2014), and *Y. divulgata* 5257/2 (isolated from grounded raw meat) (Nagy et al. 2013) grew in YPD medium for 2 days (20 g·L^−1^ glucose, 20 g·L^−1^ peptone, 10 g·L^−1^ yeast extract) were mixed with 20% (v/v) of pure glycerol and stored at −80 °C until the pre-inoculum preparation.

### Evaluation of yeast strains tolerant to LBH-C

Experiments were carried out in 96-well microplates to assess the ability of the different yeast strains to grow in a liquid medium containing each LBH-C (acetic acid, formic acid, furfural, 5-HMF, phenol, *p*-coumaric acid, vanillin and benzoic acid). Each well was filled with 30 μL of yeast culture pre-grown for 18 h in YPD medium (OD_600_ initial of 0.5), 240 μL of YPD, and 30 μL of each compound (at final concentrations in the medium of 0.1 g·L^−1^, 0.5 g·L^−1^, 1 g·L^−1^, or 5 g·L^−1^) for a total volume of 300 μL. A control experiment with only YPD was also carried out for each yeast strain. The microplates were incubated at 27 °C and 150 rpm for 48 h. The yeast growth was quantified by measuring optical density (OD_600_) at the beginning and the end of the incubation period (*Δ*OD). Results were expressed as growth inhibition (Eq. 1), where *Δ*OD_YPD_ is the yeast growth in the control experiment and *Δ*OD_LBH − C_ is the yeast growth in medium with each LBH-C.

Eq. 1 $$\textrm{Growth}\ \textrm{inhibition}=\frac{{\varDelta \textrm{OD}}_{\textrm{YPD}}-{\varDelta \textrm{OD}}_{\textrm{LBH}-\textrm{C}}}{{\varDelta \textrm{OD}}_{\textrm{YPD}}}$$

The results of the aggregated growth inhibition for each LBH-C and each yeast strain were calculated as the average of all growth inhibition values obtained for each LBH-C (across all strains and concentrations) and the average of all growth inhibition values obtained for each strain (across all LBH-C and concentrations), respectively.

### Erlenmeyer flask batch experiments

*Yarrowia lipolytica* W29 and *Y. lipolytica* NCYC 2904 batch experiments were carried out in 250-mL Erlenmeyer flasks containing 100 mL of culture medium (glucose 20 g·L^−1^ or 40 g·L^−1^, corn steep liquor 0.5 g·L^**−**1^, acetic acid 5 g·L^**−**1^, furfural 0.5 g·L^**−**1^, formic acid 0.5 g·L^**−**1^, 5-HMF 0.5 g·L^**−**1^, and ammonium sulfate for a C/N ratio of 75). An experiment without the addition of LBH-C was also carried out as a control. The pH was adjusted initially and after each sampling to 5.5 by the addition of HCl 2 M or NaOH 2 M. Yeast cells grew overnight in YPD medium and were centrifuged and resuspended in the culture medium to obtain an initial biomass concentration of 0.5 g·L^**−**1^. The Erlenmeyer flasks were placed in an orbital incubator at 27 °C and 200 rpm during 96 h (experiments with glucose 20 g·L^**−**1^) or 168 h (experiments with glucose 40 g·L^**−**1^).

### Bioreactor batch experiments

*Yarrowia lipolytica* W29 and *Y. lipolytica* NCYC 2904 batch experiments were carried out in a 3.7-L bioreactor (RALF PLUS SOLO, Bioengineering, Switzerland) filled with 1 L of culture medium (glucose 20 g·L^−1^ or 40 g·L^−1^, xylose 1 g·L^−1^, corn steep liquor 0.5 g·L^−1^, acetic acid 5 g·L^−1^, furfural 0.5 g·L^−1^, formic acid 0.5 g·L^−1^, 5-HMF 0.5 g·L^−1^, and ammonium sulfate to obtain a C/N ratio of 75). Yeast cells pre-grown overnight in YPD medium were centrifuged and resuspended in the culture medium (0.5 g·L^−1^ of initial biomass concentration). Air at 1 vvm of aeration rate was supplied with a sparger located at the bottom of the bioreactor, and the dissolved oxygen in the culture medium was measured with a polarographic probe (InPro600, Mettler Toledo, EUA) using the BioScadaLab software. The pH was measured with a probe (405-DPAS-SC-K8S, Mettler Toledo, EUA) and maintained at 5.5 by the automatic addition of HCl 2 M or NaOH 2 M, using peristaltic pump. Experiments were carried out at 27 °C and 400 rpm during 72–96 h (experiments with glucose 20 g·L^−1^) or 120–144 h (experiments with glucose 40 g·L^−1^).

### Analytical methods

Samples were collected, at defined intervals, for quantification of biomass, glucose, xylose, and LBH-C concentrations and intracellular lipid content.

Biomass concentration was quantified measuring the optical density at 600 nm and converting the absorbance to cell dry weight (g·L^−1^) using a calibration curve. Glucose, xylose, and LBH-C (acetic acid, furfural, formic acid, and 5-HMF) were measured by high-performance liquid chromatography (LC 2060C, Shimadzu, Japan) using an Aminex HPX-87H column (300 mm × 7.8 mm, 8 μm particle size), at 60 °C, coupled with RI and UV detectors. Sulfuric acid 5 mM was used in the mobile phase at a flow rate of 0.7 mL·min^−1^.

Microbial lipids were quantified in the lyophilized cells (10 mg) using the phospho-vanillin colorimetric method after extraction with a mixture of methanol and chloroform (1:1, v/v) according to Lopes et al. (2019). Briefly, the extraction mixture was vortex-mixed for 3 min, and 250-μL aliquot was collected into a test tube and heated up to 100 °C. After solvent total evaporation, 100 μL of sulfuric acid 98% was added to each tube and incubated at 100 °C for 15 min. Then, the tubes were cooled down to room temperature, 2.4 mL of phospho-vanillin reagent (vanillin in orthophosphoric acid 85%) was added, and the mixture rested for 15 min. Absorbance was read at 490 nm in a microplate reader and converted to lipids concentration (g·L^−1^) through a calibration curve (using olive oil dissolved in acetone as standard). Results were expressed as microbial lipids content (ratio between lipids concentration obtained by calibration curve and lyophilized biomass concentration used to perform the phospho-vanillin method) and microbial lipids concentration (multiplying lipids content by biomass concentration in the cultivation medium).

### Statistical analysis

One-way analysis of variance (ANOVA) was performed, and Tukey’s test was used to detect significant differences among means (*p* < 0.05). All analyses were performed in GraphPad Prism 7 software (Dotmatics, California, USA).

## Results

### Evaluation of yeast strains tolerant to LBH-C

**Fig. 1:**
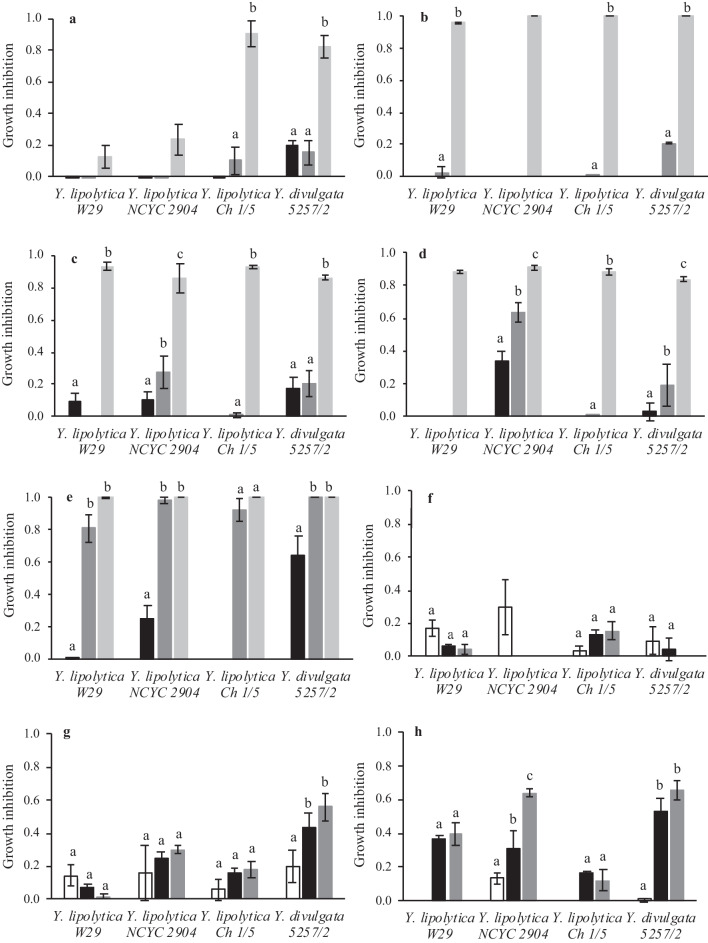
Growth inhibition of yeast strains in media with acetic acid (**a**), formic acid (**b**), furfural (**c**), 5-HMF (**d**), phenol (**e**), *p*-coumaric acid (**f**), vanillin (**g**), and benzoic acid (**h**) at different concentrations: 0.1 g·L^−1^, white bars; 0.5 g·L^−1^, black bars; 1 g·L^−1^, dark gray bars; and 5 g·L^−1^, light gray bars. The error bars represent the standard deviation of three independent replicates. Statistical analysis was individually performed for each yeast strain, and bars with the same letter are not statistically different (*p* ≥ 0.05)

Although LB pretreatments are a key step for the release of fermentable sugars, several compounds with inhibitory effects can also be formed and negatively affect yeasts growth and their metabolic functions. In this study, it was evaluated the effect of acetic acid, formic acid, furfural, 5-HMF, phenol, *p*-coumaric acid, vanillin, and benzoic acid on several *Yarrowia* strains growth (Fig. 1).

Regardless of *Y. lipolytica* strains, low concentrations of acetic acid did not affect yeasts growth (Fig. 1a). By contrast, a strong inhibitory effect was observed on *Y. lipolytica* Ch 1/5 and *Y. divulgata* 5257/2 growth when acetic acid concentration was 5 g·L^−1^. *Yarrowia lipolytica* W29 and NCYC 2904 were the most resistant strains to acetic acid, since cellular growth was only 12% and 23% lower than in YPD medium (control), respectively. Low concentrations of formic acid did not affect yeast growth (Fig. 1b), but a concentration of 5 g·L^−1^ inhibited the growth of all yeast strains. Furfural (Fig. 1c) and 5-HMF (Fig. 1d) at 5 g·L^−1^ completely inhibited the growth of all yeast strains. At low concentrations, the growth inhibition was below 21% for *Y. lipolytica* W29, *Y. lipolytica* Ch 1/5, and *Y. divulgata* 5257/2. On the other hand, *Y. lipolytica* NCYC 2904 growth in the presence of 1 g·L^−1^ of furfural and 5-HMF was 27% and 64% lower than in YPD medium, respectively, demonstrating its low tolerance for these compounds. Phenol, p-coumaric acid, vanillin, and benzoic acid are some of the phenolic compounds released during pretreatments, and different inhibition patterns were observed among them. Phenol proved to have a strong inhibitory effect since yeasts did not grow at concentrations of 1 g·L^−1^ and 5 g·L^−1^ (Fig. 1e). Phenol was particularly harmful to *Y. divulgata* 5257/2 compared to the other strains since this yeast did not grow even in the lowest concentration of phenol tested (0.5 g·L^−1^). All *Y. lipolytica* strains grew in the medium with *p*-coumaric (Fig. 1f) and vanillin (Fig. 1g) regardless of concentration tested (growth inhibition below 30%), indicating their high tolerance to these compounds. *Yarrowia divulgata* 5257/2 grew in the medium with *p*-coumaric acid but was inhibited at vanillin concentrations above 0.1 g·L^−1^, suggesting a lower tolerance to this compound. Benzoic acid had a higher inhibitory effect on yeast growth than *p*-coumaric acid and vanillin but a lower inhibition effect than phenol. The growth of *Y. lipolytica* W29, *Y. lipolytica* NCYC 2904, and *Y. divulgata* 5257/2 was significantly reduced with benzoic acid above 0.5 g·L^-1^, whereas *Y. lipolytica* Ch 1/5 was more tolerant to this compound at the same concentrations (Fig. 1h).

**Fig. 2:**
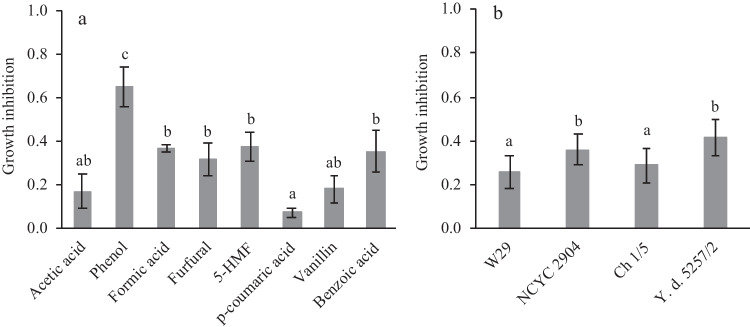
Aggregated results of growth inhibition obtained for each LBH-C (**a**) and for each yeast strain (three of *Y. lipolytica* and one of *Y. divulgata* (Y. d.)) (**b**). The error bars represent the standard deviation of the mean of all experiments. Bars with the same letter are not statistically different (*p* > 0.05)

Overall, *p*-coumaric acid, vanillin, and acetic acid were the compounds with a less inhibitory effect (below 20%) on yeast growth, whereas phenol strongly inhibited biomass propagation (Fig. 2a). Additionally, *Y. lipolytica* W29 and Ch 1/5 were the strains more tolerant to all compounds tested (Fig. 2b). These results demonstrate the potential of these *Y. lipolytica* strains to grow in LBH composed of glucose and LBH-derived compounds.

### Erlenmeyer flask batch experiments

**Fig. 3:**
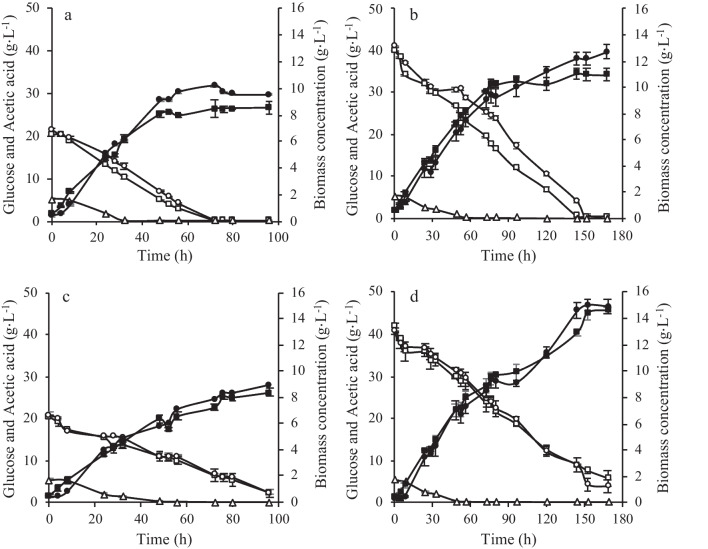
Time course of biomass production (closed symbols), glucose consumption (open symbols), and acetic acid consumption (white rectangle) obtained in *Y. lipolytica* W29 (**a**, **b**) and *Y. lipolytica* NCYC 2904 (**c**, **d**) batch cultures with 20 g·L^−1^ (left column) or 40 g·L^−1^ glucose (right column), supplemented with CSL and ammonium sulfate and with (black circle, white circle) or without (control experiments) (black square, white square) LBH-C, carried out in Erlenmeyer flasks. The error bars represent the standard deviation of two independent replicates

*Yarrowia lipolytica* W29 growth was studied in the presence of different LBH-C in Erlenmeyer flask experiments. *Yarrowia lipolytica* NCYC 2904 strain was also studied given its high ability to accumulate microbial lipids (Pereira et al. 2021; Vong et al. 2016) that is one major goal of this work. Experiments were carried out in a nitrogen-limited medium composed of glucose, LBH-C, corn steep liquor (CSL), and ammonium sulfate. Preliminary experiments demonstrated the possibility of replacing YNB (used in microplate experiments) with the low-cost CSL as nitrogen source (data not shown). Indeed, the advantages of using CSL instead of other conventional and expensive nitrogen sources were already mentioned in literature for the production of biomass and value-added products by yeasts (Kumar et al. 2017; Liu et al. 2015; Santos et al. 2013). Two concentrations of glucose (20 g·L^−1^ and 40 g·L^−1^) were studied considering the variability of sugar concentrations that occur in LBH. Regardless of glucose concentration and yeast strain, no cellular growth inhibition was observed since initial cell growth rate and final biomass production were similar or even slightly higher in the experiments with LBH-C than in their absence (Fig. 3). Indeed, the biomass yield for both yeast strains was equal in media containing LBH-C and in the control medium (Table 1). Both W29 and NCYC 2904 strains consumed all compounds, except 5-HMF that was 40 to 50% assimilated by the cells. Specifically, *Y. lipolytica* W29 assimilated all acetic acid after 32 h (20 g∙L^−1^ glucose) and 56 h (40 g∙L^−1^ glucose), furfural after 8 h (both experiments), and formic acid after 72 h (both experiments). On the other hand, similar consumption patterns were observed for NCYC 2904 strain regardless of glucose concentration, since acetic acid was completely consumed after 56 h, furfural after 8 h, and formic acid after 48 h in both conditions. Furthermore, LBH-C did not affect the glucose consumption profile (Fig. 3) and glucose uptake rate of both strains (Table 1).

**Table 1:** Values of specific growth rate (*μ*), glucose uptake rate (*GUR*), and biomass yield (*Y*_X/S_) obtained in *Y. lipolytica* W29 and *Y. lipolytica* NCYC 2904 batch cultures in complex medium containing 20 g∙L^−1^ or 40 g∙L^−1^ of glucose, with or without lignocellulosic biomass hydrolysate-derived compounds, carried out in Erlenmeyer flasks. Data are presented as average ± standard deviation of two independent replicates. Statistical analysis was performed by columns for each yeast strain, and values followed by the same letter are not significantly different (*p* ≥ 0.05)

Strain	Glucose (g∙L^−1^)	LBH-C	*μ* (h^−1^)	*GUR* (g∙L^−1^∙h^−1^)	*Y*_X/S_ (g∙g^-1^)
*Y. lipolytica* W29	20	Without	0.069 ± 0.001^a^	0.308 ± 0.003^a^	0.39 ± 0.02^a^
		With	0.087 ± 0.001^b^	0.301 ± 0.009^a^	0.35 ± 0.01^a^
	40	Without	0.065 ± 0.002^a^	0.27 ± 0.02^ab^	0.27 ± 0.02^b^
		With	0.065 ± 0.005^a^	0.24 ± 0.01^b^	0.27 ± 0.02^b^
*Y. lipolytica* NCYC 2904	20	Without	0.08 ± 0.01^a^	0.18 ± 0.01^a^	0.42 ± 0.03^a^
		With	0.11 ± 0.01^ab^	0.19 ± 0.03^a^	0.34 ± 0.02^a^
	40	Without	0.085 ± 0.001^a^	0.23 ± 0.01^a^	0.39 ± 0.03^a^
		With	0.128 ± 0.002^b^	0.22 ± 0.02^a^	0.33 ± 0.02^a^

*GUR* was calculated by the slope of glucose concentration over the whole time of the experiment till total glucose consumption; *μ* was calculated by the slope of the logarithmic growth function in the exponential growth phase; *Y*_X/S_ was calculated at 96 h (20 g∙L^−1^ glucose) or 168 h (40 g∙L^−1^ glucose) considering the consumption of glucose and acetic acid

Besides biomass production, microbial lipids accumulation was also assessed during *Y. lipolytica* W29 and NCYC 2904 growth. No negative effects were observed on microbial lipids accumulation by the mixture of LBH-C tested. In fact, lipids concentration was highest in LBH-C containing media regardless of the glucose concentration (Table 2), being more evident for NCYC 2904 strain. *Yarrowia lipolytica* NCYC 2904 accumulated more intracellular lipids than the W29 strain, particularly in the experiments carried out with 40 g∙L^−1^ of glucose. In these conditions, lipids concentration attained by NCYC 2904 strain was fourfold and fivefold higher than those obtained by W29 strain in medium with and without LBH-C, respectively (Table 2).

**Table 2:** Values of microbial lipids content and microbial lipids concentration obtained at the end of *Y. lipolytica* W29 and *Y. lipolytica* NCYC 2904 batch cultures in complex medium containing 20 g∙L^−1^ or 40 g∙L^−1^ of glucose, with or without lignocellulosic biomass hydrolysate-derived compounds, carried out in Erlenmeyer flasks. Data are presented as average ± standard deviation of two independent replicates. Statistical analysis was performed by columns for each yeast strain, and values with the same letter are not significantly different (*p* ≥ 0.05)

Strain	Glucose (g∙L^−1^)	LBH-C	Lipids content (%, w/w)	Lipids concentration (g∙L^-1^)
*Y. lipolytica* W29	20	Without	10.1 ± 5.6^a^	0.9 ± 0.5^a^
		With	15.8 ± 1.1^a^	1.5 ± 0.1^a^
	40	Without	7.50 ± 0.7^a^	0.8 ± 0.1^a^
		With	7.1 ± 1.4^a^	0.9 ± 0.3^a^
*Y. lipolytica* NCYC 2904	20	Without	21.4 ± 2.2^a^	1.8 ± 0.1^a^
		With	24.2 ± 0.1^ab^	2.17 ± 0.04^b^
	40	Without	23.7 ± 1.1^ab^	3.4 ± 0.1^c^
		With	28.6 ± 2.4^b^	4.2 ± 0.1^d^

### Bioreactor experiments

Since *Y. lipolytica* W29 and NCYC 2904 grew and produced lipids on media containing glucose and LBH-C, the process was scaled up from Erlenmeyer flasks to a lab-scale stirred tank bioreactor (STR), in which pH was controlled through the operation time and oxygen mass transfer was higher than in the Erlenmeyer flasks. Xylose was also added in these experiments since this sugar is also produced during LB pretreatments and is important to evaluate the yeast’s ability to consume xylose and its potential impact on the yeast’s performance.

**Fig. 4:**
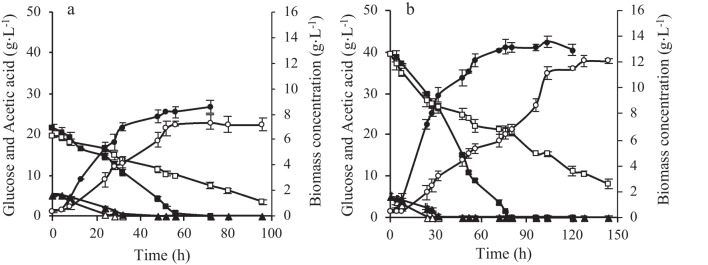
Time course of biomass production (black circle, white circle), glucose consumption (black square, white square), and acetic acid consumption (black rectangle, white rectangle) obtained in *Y. lipolytica* W29 (closed symbols) and *Y. lipolytica* NCYC 2904 (open symbols) batch cultures with LBH-C, xylose, and 20 g·L^−1^ of glucose (**a**) or 40 g·L^−1^ of glucose (**b**) carried out in an STR lab-scale bioreactor. The error bars represent the standard deviation of two independent replicates

In the bioreactor, it was possible to reproduce the results obtained in Erlenmeyer flask experiments. The final biomass concentration (Fig. 4) and the biomass yield (Table 3) of *Y. lipolytica* W29 cultures were similar, whereas the maximum specific growth rate (for both glucose concentrations) was highest in bioreactor experiments. The glucose uptake rate was 1.2-fold and 2.2-fold higher in 20 g·L^−1^ and 40 g·L^−1^ glucose-containing media, respectively, compared to Erlenmeyer flasks, being the period of glucose consumption reduced from 72 to 56 h and from 152 to 80 h, respectively. Regarding *Y. lipolytica* NCYC 2904 experiments, although the final biomass obtained in the bioreactor was similar to that obtained in Erlenmeyer flasks (for both glucose concentrations), the specific growth rate was lower in these experiments. Furthermore, glucose uptake rate and biomass yield were statistically equal in bioreactor and Erlenmeyer flask experiments, for both glucose concentrations (Table 3). In *Y. lipolytica* NCYC 2904 bioreactor experiments, the presence of glucose in the culture medium was also observed at the end of the experiments, similar to the Erlenmeyer flasks.

**Table 3:** Values of specific growth rate (*μ*), glucose uptake rate (*GUR*), and biomass yield (*Y*_X/S_) obtained in *Y. lipolytica* W29 and *Y. lipolytica* NCYC 2904 batch cultures in complex medium containing lignocellulosic biomass hydrolysate-derived compounds, xylose, and 20 g·L^−1^ or 40 g·L^−1^ of glucose and carried out in an STR lab-scale bioreactor. Data are presented as average ± standard deviation of two independent replicates. Statistical analysis was performed by columns, and values with the same letter are not significantly different (*p* ≥ 0.05)

**Glucose (g∙L**^**−1**^**)**	**Strain**	***μ*** **(h**^**−1**^**)**	***GUR*** **(g∙L**^**−1**^**∙h**^**−1**^**)**	***Y***_**X/S**_ **(g∙g**^**−1**^**)**
20	W29	0.10 ± 0.01^a^	0.36 ± 0.01^a^	0.35 ± 0.04^ab^
	NCYC 2904	0.063 ± 0.001^b^	0.17 ± 0.02^b^	0.40 ± 0.01^ab^
40	W29	0.107 ± 0.003^a^	0.531 ± 0.001^c^	0.28 ± 0.01^b^
	NCYC 2904	0.041 ± 0.001^b^	0.192 ± 0.004^b^	0.33 ± 0.01^ab^

*GUR* was calculated by the slope of glucose concentration over the whole time of the experiment till total glucose consumption; *μ* was calculated by the slope of the logarithmic growth function in the exponential growth phase; *Y*_X/S_ was calculated at 72 h (20 g∙L^−1^ glucose) or 120 h (40 g∙L^−1^ glucose) considering the consumption of glucose and acetic acid

Both yeast strains consumed LBH-C, as observed in flask experiments. In the bioreactors, regardless of the initial glucose concentration, acetic acid was completely consumed after 28 h and 48 h by *Y. lipolytica* W29 and NCYC 2904, respectively. Furfural was totally assimilated after 8 h, while 52–70% of 5-HMF was consumed. Different consumption patterns of formic acid were observed for each strain. While it was totally consumed by *Y. lipolytica* NCYC 2904 (faster in 20 g∙L^−1^ glucose experiments), *Y. lipolytica* W29 assimilated 50% of formic acid. Regardless of yeast strain and initial glucose concentration, xylose was not consumed throughout the cultivation time. Even in the experiments with the W29 strain, in which glucose was no longer available in the culture medium after 56 h (20 g∙L^−1^ glucose) or 80 h (40 g∙L^−1^ glucose), xylose was not assimilated.

**Fig. 5:**
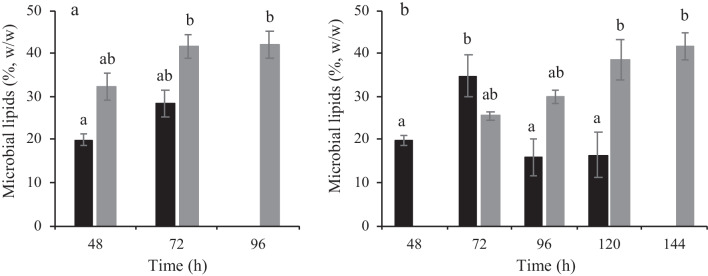
Microbial lipids content of *Y. lipolytica* W29 (black bars) and *Y. lipolytica* NCYC 2904 (gray bars) cells obtained in batch cultures with LBH-C, xylose, and 20 g·L^−1^ (**a**) or 40 g·L^−1^ of glucose (**b**) carried out in an STR lab-scale bioreactor. The error bars represent the standard deviation of two independent replicates

*Yarrowia lipolytica* W29 and NCYC 2904 also synthesized intracellular lipids in the bioreactor experiments, and higher lipids content and concentration were reached compared to the flask experiments. Bioreactor experiments led to a 2.4-fold and 1.5-fold improvement in maximum lipids content accumulated by *Y. lipolytica* W29 and NCYC 2904, respectively (Fig. 5).

Lipids content increased during experiments using 20 g∙L^−1^ glucose, reaching 28% (w/w) and 42% (w/w) for W29 and NCYC 2904 strains, respectively. Lipids accumulation showed two different patterns for each strain in glucose 40 g∙L^−1^ experiments: (a) for *Y. lipolytica* W29, lipid content reached 35% (w/w) after 72 h, decreasing afterwards, and (b) in *Y. lipolytica* NCYC 2904, lipids content continuously increased during the experiment achieving 39% (w/w) after 120 h (Fig. 5). The lipids accumulated by the W29 strain were mobilized after 72 h (lipid turnover) due to the depletion of carbon sources in the culture medium (the maximum value corresponds to the beginning of the stationary growth phase and the depletion of glucose and LBH-C).

As observed in the Erlenmeyer flask experiments, the intrinsic ability of NCYC 2904 strain to accumulate more lipids than W29 strain was also verified in bioreactor experiments. In general, the maximum lipids content accumulated by NCYC 2904 strain from glucose was slightly higher than those attained by W29 strain, despite the difference was not statistically significant (Fig. 5). However, *Y. lipolytica* NCYC 2904 produced 4.5 g∙L^−1^ (20 g∙L^−1^ glucose) and 5.0 g∙L^−1^ (40 g∙L^−1^ glucose) of lipids, corresponding to a 1.7-fold and 1.6-fold improvement relative to lipids concentration attained by W29 strain.

## Discussion

In microplate experiments, it was proved the high resistance to acetic acid by two strains (W29 and NCYC 2904) (Fig. 1a). This compound is formed during the hydrolysis of acetyl groups of hemicellulose, and it can diffuse inside the cell and dissociate in acetate owing to the alkaline pH of cytoplasm, causing intracellular accumulation of anions and acidification (Almeida et al. 2007; Konzock et al. 2021; Santek et al. 2018). Cell viability and biomass formation can be affected by the acetic acid concentration and intracellular acidification since these conditions cause oxidative damage and ATP depletion (protons are pumped out of the cell) (Guaragnella and Bettiga 2021). Although acetic acid may be a stress factor for yeasts, moderate concentrations of this acid can be used as a regular carbon source (Guaragnella and Bettiga 2021). As observed in this work, some authors reported the ability of *Y. lipolytica* strains to use up to 110 g·L^−1^ of acetic acid (or its dissociated form acetate) as a carbon source for biomass and microbial lipids production (Gao et al. 2020; Miranda et al. 2020; S. Pereira et al. 2021). Acetate can be converted into acetyl-CoA (by the acetyl-CoA synthetase), which is either transformed into different lipids in the endoplasmic reticulum, enter the mitochondrial tricarboxylic acid cycle, or be converted to succinate by the glyoxylate cycle (Qin et al. 2017).

Formic acid is generated when furfural and 5-HMF are exposed to high temperatures in an acidic environment for long periods (Santek et al. 2018). Despite having the same inhibition mechanism as acetic acid, formic acid exhibited higher toxicity in all studied strains (Fig. 1b), which may be explained by the lower pKa value (3.75 at 25 °C) compared to acetic acid (4.75 at 25 °C) (Almeida et al. 2007). Thus, for the same concentration, formic acid results in a higher decrease of intracellular pH compared to acetic acid. Additionally, formic acid diffuses more easily through the plasmatic membrane given its lower molecular size (Konzock et al. 2021). Similar to the results observed in this study, Konzock et al. (2021) and Guo and Olsson (2014) observed higher toxicity of formic acid than acetic acid on *Y. lipolytica* and *S. cerevisiae* growth.

The aldehydes furfural and 5-HMF are formed through the dehydration of pentoses and hexoses released during hemicellulose and cellulose hydrolysis, respectively (Almeida et al. 2007). Furfural and 5-HMF are commonly identified as the most potent inhibitor compounds found in LBH since they reduce microorganisms’ enzymatic and biological activities, compromising the glycolytic activity and the tricarboxylic acid cycle, damaging the DNA, and inhibiting protein and RNA synthesis (Almeida et al. 2007; Iwaki et al. 2013; Liu et al. 2004). Nonetheless, some yeasts may resist, at least until a certain extent, to the presence of these and other inhibitory-like compounds, as was observed for W29 and Ch 1/5 strains (up to 1 g·L^−1^ of furfural (Fig. 1c) or 5-HMF (Fig. 1d)). *Saccharomyces cerevisiae* does not metabolize furfural and 5-HMF but it can grow, at least to some extent, in their presence since it possesses non-specific dehydrogenases and reductases that oxidize or reduce furfural and 5-HMF into less inhibitory compounds, such as alcohols or acids (Taherzadeh et al. 1999). According to Konzock et al. (2021), *Y. lipolytica* seems to have the same mechanism to detoxify these compounds. However, this is not always observed, since a previous study demonstrated that *Y. lipolytica* CBS 1073 was not tolerant to 0.5 g·L^−1^ of furfural (Sitepu et al. 2014). Indeed, the results reported herein support that furfural tolerance is dependent on yeast strain (Fig. 1c). Compared to 5-HMF, some studies reported a higher inhibitory effect of furfural on *Y. lipolytica* and other yeast species growth (Almeida et al. 2007; Caporusso et al. 2021; Sitepu et al. 2014), which was not observed in this study at the concentrations used (Fig. 1d).

The formation of phenolic compounds derived from lignin and extractive compounds is strongly dependent on the type of pretreatment, processing conditions, and LB origin (Almeida et al. 2007; Santek et al. 2018). There are many studies comparing the inhibitory effect of different phenolic acids in yeast growth, but the results are not consensual. In fact, different inhibition patterns were attained for the four phenolic compounds tested (Fig. 1). Some authors stated that the harmful effects of phenolic compounds on yeast growth are due to damages inflicted on the biological membranes (loss of membrane integrity and electrochemical gradient change) (Yu et al. 2014). Negritto et al. (2017) assigned phenolic compound toxicity to their hydrophobicity and/or the formation of free radicals. It was also reported that phenol and other benzene-like compounds may significantly damage DNA, whereas other phenolic compounds protect DNA from other harmful factors. Although benzoic acid is typically classified as a phenolic compound found in LBH, its inhibition mechanism is similar to that of weak acids such as acetic and formic acids (Verduyn et al. 1992). In accordance with those observed for strains studied (Fig. 1e), Dias et al. (2021) also noticed that other *Y. lipolytica* strains were not able to grow in catechol, tyrosol, and phenol at 1 g·L^−1^. Jarboui et al. (2012) observed that 1 g·L^−1^ of *p*-coumaric and vanillic acids did not inhibit *Rhodotorula mucilaginosa* growth. Vanillin was more harmful to *R. toruloides* Y4 growth than acetate, 5-HMF, and syringaldehyde (Hu et al. 2009). The addition of benzoic acid to a glucose medium decreased biomass yield and the specific oxygen uptake rate of *Saccharomyces cerevisiae* CBS 8066, *Candida utilis* CBS 621, *Hansenula polymorpha* ATCC 46059, and *Kluyveromyces marxianus* CBS 6556 (Verduyn et al. 1992).

In Erlenmeyer flask batch experiments, the absence of yeast growth inhibition was expected since the LBH-C, at the same concentrations, did not negatively affect yeast growth in the microplate experiments. Furthermore, the final biomass was slightly higher in the experiments with LBH-C than in their absence, indicating that yeasts used the additional compounds as carbon source (Fig. 3). The toxic effect of LBH-C on yeast growth depends on yeast strains and the type and concentration of these compounds (Almeida et al. 2007). Some authors reported that the presence of several LBH-C may have synergistic or antagonistic effects on microbial metabolism. Konzock et al. (2021) suggested that the simultaneous addition of furfural, coniferyl aldehyde, and formic acid exerts an excessive pressure on the cell, resulting in the overproduction of reactive oxygen species (ROS). Formic acid, acetic acid, furfural, and vanillin had a negative effect on biomass production and glucose and xylose consumption by *Rhodotorula toruloides* (Hu et al. 2009). On the other hand, low concentrations of acetic acid (20 mM) improved *S. cerevisiae* tolerance to 5-HMF and furfural, favoring glucose utilization and ethanol production (Greetham et al. 2016). The current work proves that *Y. lipolytica* strains are highly resistant to the mixture of LBH-C tested, since synergistic effects among compounds were not observed. Then, these results indicate that growing these strains in LBH do not require detoxification steps, being economically advantageous in real lignocellulosic hydrolysates.

The non-conventional yeast *Y. lipolytica* is considered an oleaginous yeast, due to its high ability to accumulate intracellular lipids using several pure and unrefined raw materials (Lopes et al. 2021). In hydrophilic substrates (e.g. glucose, acetic acid), de novo lipids synthesis occurs during the stationary growth phase (oleaginous phase), in which intracellular lipids accumulation is favored in conditions of carbon excess and nitrogen limitation (Lopes et al. 2021). These substrates can be metabolized to acetyl-CoA by *Y. lipolytica* and enter in de novo lipids synthesis pathway. After carboxylation of acetyl-CoA to malonyl-CoA (by acetyl-CoA carboxylase), multiple cyclic series of elongation (by fatty acid synthetases) occur, resulting in different fatty acyl-CoAs or phospholipids with variable carbon chain lengths. These compounds are then converted into various lipids, such as triglycerides and steryl esters (Mahajan et al. 2019).

It was reported that some LBH-C may repress the microbial lipids accumulation pathway (Zainuddin et al. 2022). Poontawee et al. (2017) concluded that formic acid and furfural strongly inhibited *Rhodotorula fluviale* lipids accumulation, while the acetic acid, 5-HMF, and vanillin had little effect on lipids content. However, other studies suggest that these compounds at low/moderate concentrations only slow down cell growth, while microbial lipids accumulation is unaffected (Hu et al. 2009). In this work, the higher lipids accumulation in experiments with LBH-C than in their absence (Table 2) may be explained by the highest availability of non-inhibitory and assimilable carbon sources for yeasts (namely 5 g∙L^−1^ of acetic acid). The simultaneous consumption of acetic acid and glucose possibly increased the proportion of acetyl-CoA in the cytosol, increasing lipids concentration since it is directly utilized in fatty acid biosynthesis. It was already observed that the addition of acetic acid as a co-substrate of glucose enhanced lipids production by *Cryptococcus curvatus* (Gong et al. 2016) and *Trichosporon cutaneum* (Chen et al. 2009). There are some studies indicating that there is an increase in lipids production when acetic acid is a co-substrate (along with glucose, other volatile fatty acids, glycerol, or xylose) on various oleaginous yeasts, such as *Y. lipolytica* (Pereira et al. 2021), *C. curvatus* (Gong et al. 2016), and *R. toruloides* (Chmielarz et al. 2021).

Operational conditions such as pH-controlled and oxygenation are determinant factors to successfully scale-up a bioprocess. In fact, *Y. lipolytica* growth and microbial lipids accumulation are strongly influenced by the dissolved oxygen concentration in the cultivation medium (Magdouli et al. 2018). In the current work, the results suggest that NCYC 2904 strain may require lower oxygenation conditions than that used in the bioreactor experiments, contrary to those observed for W29 strain. Therefore, it is possible to conclude that, although final biomass was similar for both strains (Fig. 4), bioreactor experiments proved to be more advantageous for *Y. lipolytica* W29 growth since the maximum biomass productivity was higher than for NCYC 2904 strain.

In the literature, many studies reported *Y. lipolytica* inability to grow on xylose as the sole carbon source without resorting to genetic engineering (Yao et al. 2020). However, a cryptic xylose utilization pathway and xylose-degrading enzymes were discovered in the last years in the *Y. lipolytica* genome (Drzymała-Kapinos et al. 2022; Rodriguez et al. 2016), and some reports attribute the capacity of the Po1g strain to grow in xylose (Ledesma-Amaro et al. 2016). Nevertheless, *Y. lipolytica* growth in xylose is stated as insufficient without an adaptation phase or genetic modification (Drzymała-Kapinos et al. 2022). In flask batch cultures, yeast strains studied in this work (W29 and NCYC 2904) grew in a medium containing 1 g∙L^−1^ xylose as sole carbon source. Furthermore, in a medium containing a mixture of 50 g∙L^−1^ glucose and 8 g∙L^−1^ xylose, both yeast strains demonstrated a preference for glucose (unpublished results). This sequential metabolization of sugars was already reported for other yeasts and has been explained either as a mechanism of catabolite repression caused by glucose or due to an allosteric competition for sugars transporter (Poontawee et al. 2017). Nonetheless, in this study, xylose concentration, the experiment duration, and experimental conditions used in the bioreactor may have not be sufficient to trigger the xylose utilization pathway in both strains.

Some process variables such as mechanical agitation, pH-controlled, forced aeration, and bioreactor configuration possibly contributed to higher lipids production in bioreactor experiments (Fig. 5) compared to flask experiments (Table 2). In the case of strictly aerobic yeasts such as *Y. lipolytica*, high mechanical agitation rate and forced aeration are particularly important to increase the volumetric mass transfer coefficient (*k*_L_a), resulting in an improvement of oxygen mass transfer rate (OTR) from the gas phase to the liquid medium (Lopes et al. 2014). Generally, lipids accumulation by *Y. lipolytica* strains increases in highly aerated cultures using glucose as carbon source (Bellou et al. 2014). Magdouli et al. (2018) reported that high concentrations of dissolved oxygen (up to certain levels) upregulated enzymes activity involved in lipids synthesis (i.e., ATP-citrate lyase and Malate dehydrogenase), improving lipid production.

The lipids content decrease (lipid turnover) observed in 40 g∙L^−1^ glucose experiments with *Y. lipolytica* W29 in the final stage of growth (Fig. 5) was already demonstrated in other studies (Sarris et al. 2017, 2011) and is possibly explained by the depletion of the carbon source or with the uptake repression observed when the carbon source amount does not meet the cells metabolic requirements (Magdouli et al. 2018).

The intrinsic ability of NCYC 2904 strain to accumulate more lipids than W29 strain verified in Erlenmeyer flask (Table 2) and bioreactor experiments (Fig. 5) was also reported by Pereira et al. (2021). Intracellular lipids accumulated by *Y. lipolytica* NCYC 2904 in a medium containing 20 g∙L^−1^ glucose and 6 g∙L^−1^ volatile fatty acids (acetate, propionate, and butyrate) was 45% higher than that obtained with W29 strain. Nevertheless, although this different ability, both yeast strains studied are suitable to produce microbial lipids. To the best of our knowledge, the production of microbial lipids by *Y. lipolytica* wild strains from LBH at the bioreactor scale is still few explored. Yet, the lipids content obtained herein is higher than that reported for *Y. lipolytica* CBS7504 growing in switchgrass hydrolysate (18%, w/w) (Walker et al. 2021). It is also important to highlight that the maximum lipids content (42%, w/w) produced by a mutant xylose-utilizing *Y. lipolytica* strain in miscanthus hydrolysate (Yook et al. 2020) is equal to those attained in this study by wild-type strains. Niehus et al. (2018) demonstrated that engineered *Y. lipolytica* strains were able to grow and produce 67% (w/w) of microbial lipids on agave bagasse hydrolysate in batch followed by fed-batch operation mode.

In summary, the results described herein demonstrate the high ability of *Y. lipolytica* W29 and NCYC 2904 strains to grow in the presence of compounds commonly found in lignocellulosic biomass hydrolysates, which are often associated with inhibitory effects on microbial growth. In STR lab-scale bioreactor, *Yarrowia lipolytica* W29 and NCYC 2904 strains accumulated, respectively, 35% and 42% (w/w) of intracellular lipids in a synthetic medium containing glucose, xylose, acetic acid, formic acid, furfural, and 5-HMF. These results demonstrate the potential of using lignocellulosic hydrolysates, without detoxification steps to remove undesirable compounds, for yeast growth and microbial lipids production. Future research should focus on the optimization of operational conditions (pH, agitation rate, aeration rate) in bioreactors aiming to achieve high microbial lipids production using real lignocellulosic biomass hydrolysates. Following a circular bioeconomy approach to foment sustainable practices, the present work is a significant first step towards microbial lipids production using *Y. lipolytica* strains by reutilizing abundantly generated lignocellulosic biomass.

## Data Availability

The datasets generated during and/or analyzed during the current study are available from the corresponding author on a reasonable request.
